# Molecular Interactions between (−)-Epigallocatechin Gallate Analogs and Pancreatic Lipase

**DOI:** 10.1371/journal.pone.0111143

**Published:** 2014-11-03

**Authors:** Shihui Wang, Zeya Sun, Shengzhao Dong, Yang Liu, Yun Liu

**Affiliations:** Beijing Key Laboratory of Bioprocess, The Biorefinery Research and Engineering Center of the Ministry of Education of China, College of Life Science and Technology, Beijing University of Chemical Technology, Beijing, China; Weizmann Institute of Science, Israel

## Abstract

The molecular interactions between pancreatic lipase (PL) and four tea polyphenols (EGCG analogs), like (−)-epigallocatechin gallate (EGCG), (−)-gallocatechin gallate (GCG), (−)-epicatechin gallate (ECG), and (−)-epigallocatechin (EC), were studied from PL activity, conformation, kinetics and thermodynamics. It was observed that EGCG analogs inhibited PL activity, and their inhibitory rates decreased by the order of EGCG>GCG>ECG>EC. PL activity at first decreased rapidly and then slowly with the increase of EGCG analogs concentrations. α-Helix content of PL secondary structure decreased dependent on EGCG analogs concentration by the order of EGCG>GCG>ECG>EC. EGCG, ECG, and EC could quench PL fluorescence both dynamically and statically, while GCG only quenched statically. EGCG analogs would induce PL self-assembly into complexes and the hydrodynamic radii of the complexes possessed a close relationship with the inhibitory rates. Kinetics analysis showed that EGCG analogs non-competitively inhibited PL activity and did not bind to PL catalytic site. DSC measurement revealed that EGCG analogs decreased the transition midpoint temperature of PL enzyme, suggesting that these compounds reduced PL enzyme thermostability. *In vitro* renaturation through urea solution indicated that interactions between PL and EGCG analogs were weak and non-covalent.

## Introduction

Obesity, mainly resulting from the excessive intake of calorie, has rapidly become a worldwide epidemic. Modern obesity epidemic not only impairs the visual appearance of body, but also induces many syndromes like diabetes, cancer, cardiovascular disease, hypertension and hyperlipidemia [1]–[3]. Triglyceride with the characteristics of high calories is the major energy source of modern humans, therefore inhibition the uptake of triglyceride is regarded as one of the most important therapies to prevent obesity and obesity-related diseases [4]. Triglyceride is hardly directly absorbed by human intestine unless it has been hydrolyzed by pancreatic lipase (PL). So PL (E.C. 3.1.1.3), also known as triacylglycerol hydrolase, is crucial for the uptake of lipid and the inhibition of PL activity can effectively reduce the triglyceride intake [5], [6]and prevent obesity to some content.

**Figure 1:**
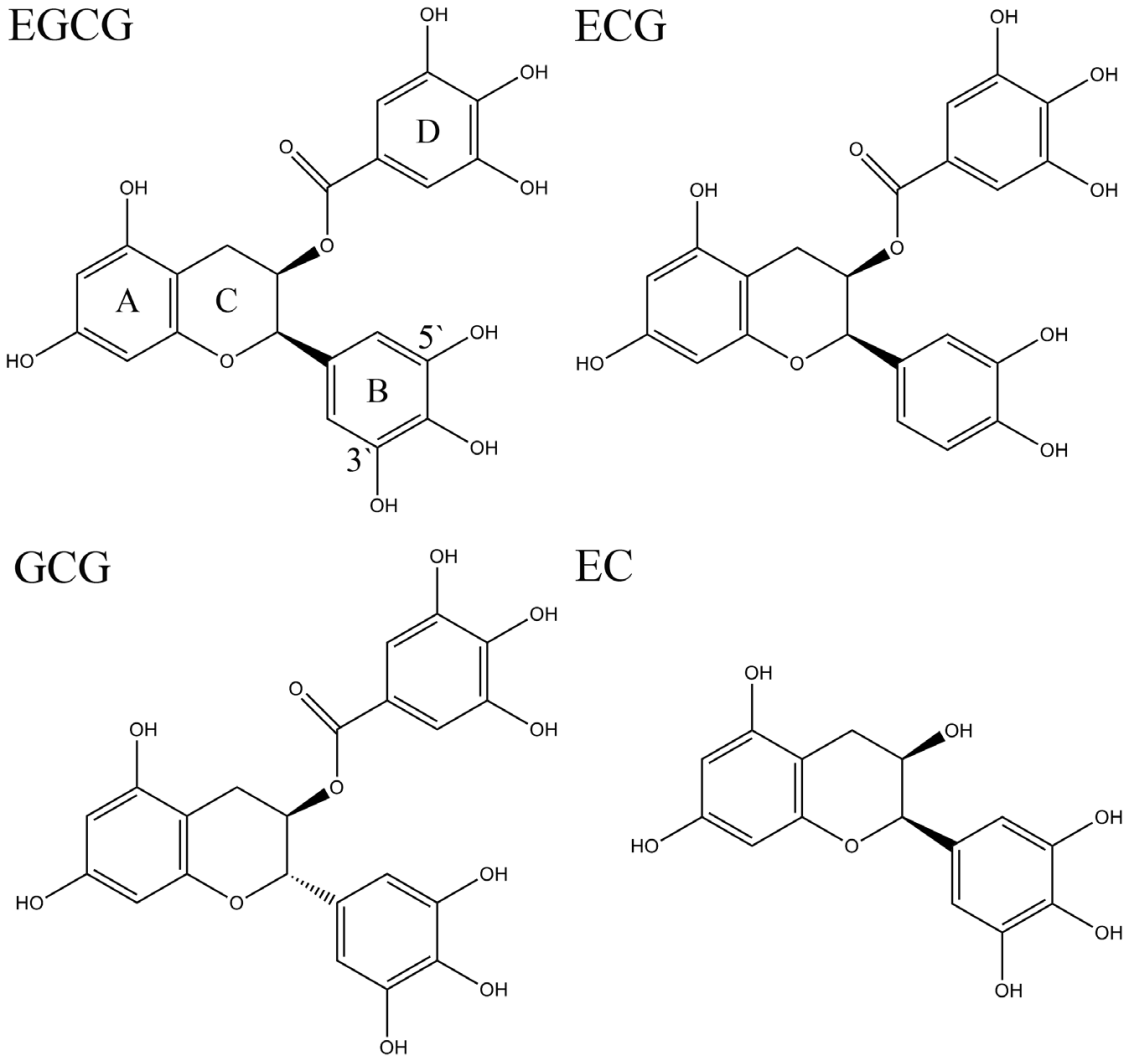
The chemical structures of EGCG analogs.

Recently, numerous inhibitors against PL activity have been explored and the natural compounds have been receiving a world-wide attention due to their excellent inhibitory effects and low toxic effects [7]–[9]. Among the natural compound inhibitors of PL, green tea polyphenols are increasingly taken into consideration owing to their anti-obesity, anti-cancer, anti-tumor, anti-inflammation, antivirus, and neuroprotective properties [10]–[13]. Bose et al. found that (−)-epigallocatechin gallate (EGCG), the major active ingredient of green tea polyphenols, could inhibit obesity, metabolic syndrome, and fatty liver disease in high-fat-fed mice [14]. Sergent et al. reported that EGCG inhibited the PL activity with an *IC*_50_ value of 0.8 µmol/L [15]. Grove et al. found that EGCG inhibited the PL activity with an *IC*_50_ value of 7.5 µmol/L in a non-competitive manner and reduced body weight in high-fat-fed obese mice [16]. Nakai et al. measured the *IC*_50_ values of 54 tea polyphenols for their inhibitory activities against PL. The authors found that the *IC*_50_ values of EGCG with one galloyl group, (–)-epigallocatechin 3, 5-digallate with two galloyl groups and (−)-epigallocatechin (EC) with no galloyl group were 0.349, 0.098, and 20 µmol/L, respectively [17]. These results indicated that the galloyl group might be of importance for the inhibition of PL activity. As depicted in Figure 1, compared to EGCG, (−)-epicatechin gallate (ECG) does not contain 3′-hydroxyl group in the structure, (−)-epigallocatechin (EC) does not include the gallol moiety, and (−)-gallocatechin gallate (GCG) is an epimer of EGCG. However, the mechanisms of interaction between the gallol and hydroxyl groups of EGCG and PL are still unclear.

Herein, we applied circular dichroism (CD) spectroscopy, fluorescence spectroscopy (FS), dynamic light scattering (DLS), and differential scanning calorimetry (DSC) approaches to characterize the effect of gallol moiety and 3′-hydroxyl group in the structure of EGCG analogs on the interactions between EGCG analogs and PL. We also investigated the binding patterns of epimers EGCG and GCG to the PL protein. More specifically, we conducted research in the following four aspects to achieve our goals: (1) to explore the influences of EGCG analogs on the activity of PL as functions of incubation time and EGCG analogs concentration using oliver oil as a substrate; (2) to investigate the impacts of EGCG analogs on the secondary and tertiary strucdtures of PL via far-UV circular dichroism (CD) and fluorescence spectroscopes (FS); (3) to evaluate the effects of EGCG analogs on the hydrodynamic radius (*R*_h_) of PL by dynamic light scattering (DLS) and the transition midpoint temperatures (*T*_m_) of PL by differential scanning calorimetry (DSC); (4) to determine the kinetic parameters of *K*_m_ and *V*_max_ from Lineweaver-Burk plots and examine the inhibitory types of EGCG analogs.

## Materials and Methods

### 1. Materials and r parameter determination

PL from porcine pancreas type II was purchased from Sigma (St. Louis, MO, USA) with a hydrolysis activity of 351 U/mg-protein (using olive oil as substrate and incubating for 30 min as reported by the manufacturer). The protein content obtained by Bradford assay is 5.21±0.26% (m/m) [18]. PL was purified by a 48-h dialysis before usage to insure the purity of PL (>95%). EGCG analogs with a purity of 98% were bought from Shanghai Yuanye Biological Technology Co., Ltd (Shanghai, China). Olive oil was purchased from Terra Delyssa (Shanghai, China). Other chemicals and agents were all of the analytic grades and bought from local markets. The buffer used was 100 mmol/L phosphate buffer solutions (PBS) (pH 7.4). To maintain low oxygen partial pressure and suppress the oxidation of EGCG, PBS was degassed for 20 min before use and the solutions were flushed with nitrogen during operations. Under this condition, the recovery of EGCG was about 96% after 2-h incubation at 37°C (see Figure S1 in File S1). The concentrations of PL and EGCG analogs we used were 2.5 µmol/L and 0–1 mmol/L. Within the range, no insoluble aggregates were observed in the PL-EGCG analogs mixture (see Table S1 in File S1).

To facilitate the comparison of different EGCG analogs under various conditions, *r* representing the molar ratio of a certain EGCG analogs to PL was introduced in this work, which was calculated as follows:
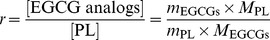
(1)where: [EGCG analogs] and [PL] are the final concentrations of a certain EGCG analogs and PL after mixing (mmol/L), respectively. *m*_EGCG analogs_ and *m*_PL_ are the mass concentration of EGCG analogs and PL protein (mg/mL), respectively. *M*_EGCG analogs_ and *M*_PL_ are the molecular weight of EGCG analogs and PL (the molecular weights are: EGCG, 458.14 g/mol; GCG, 458.14 g/mol; ECG, 442.37 g/mol; EC, 306.27 g/mol; PL, 52 kD) [4].

### 2. PL activity assays

PL activity assay was modified according to the method reported in our previous work [18]. Briefly, PL (2.5 µmol/L) and EGCG analogs (0–1 mmol/L) were dissolved in 100 mmol/L PBS (pH 7.4) and incubated at 37°C for a certain time. Then the resulting solution was mixed with 25% homogenized olive oil (v/v) (substrate) and incubated at 37°C for 30 min. The released fatty acid was titrated with 0.05 mol/L sodium hydroxide using phenolphthalein as the indicator to obtain the molar mass of it. The reaction mixture without the enzyme was titrated as the blank control. One ‘lipase unit’ was defined as the amount of the enzyme that released 1 µmol free fatty acid per minute under standard assay conditions. The inhibitory rate of EGCG analogs was expressed as Eq. 2:
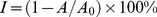
(2)where: *I* is the inhibitory rate of a certain EGCG analogs on the PL activity, *A*_0_ and *A* are the PL activities with or without EGCG analogs, respectively.

### 3. Far-UV circular dichroism analysis

The secondary structure of PL was determined by a Jasco 810 circular dichroism spectrophotometer (Jasco Inc., Tokyo, Japan) according to the method described in our previous work [19]. Briefly, PL (2.5 µmol/L) and EGCG analogs (0–1 mmol/L) were mixed and incubated at 37°C for 45 min. The sample was centrifuged at 5000 rpm for 15 min. The supernatant was diluted 2.5 times and injected into a 1-mm path length quartz cuvette. A background CD spectrum of buffer solution was subtracted from the sample spectrum for baseline correction. Spectra were recorded under the conditions: a resolution of 0.5 nm, scanning rate of 100 nm/min, response time of 1 s, bandwidth of 2 nm, room temperature and the wavelength ranges from 250 to 190 nm. The PL secondary element percentages of α-helix, β-sheet, turn, and unordered coil were calculated by spectra data using SELCON3 website online [20].

### 4. Fluorescence spectroscopy measurement

The effects of EGCG analogs on the tryptophan fluorescence spectra of PL were obtained on a Varian Cary Eclipse fluorescence spectrometer (Varian Inc., Palo Alto, California, USA) according to the method detailed in our previous work [21]. Aliquot of 2.5 µmol/L PL solution in the absence or presence of EGCG analogs (0–1 mmol/L) was incubated at 37°C for 45 min. Then the sample was centrifuged at 5000 rpm for 15 min and the supernatant was injected into a 1 cm-path length quartz cuvette. The excitation wavelength was 295 nm, and the intrinsic fluorescence was recorded from 300 to 500 nm. The excitation and emission slits were 5 and 10 nm, respectively. The scanning rate was 600 nm/min and the resolution was 1.0 nm.

The fluorescence quenching is usually described by the linear Stern-Volmer equation [22]: 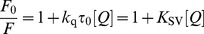
(3)where: *F*_0_ and *F* are the fluorescence intensities in the absence and presence of the quencher (herein refers to EGCG analogs), respectively. 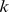
_q_ is the bimolecular quenching constant, *τ*_0_ is the lifetime of the fluorescence in the absence of the quencher, and [Q] is the concentration of the quencher. *K*_SV_, the Stern-Volmer quenching constant, is calculated by 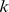
_q_ *τ*_0_, where *τ*_0_ is equal to 1.59 ns [23]. A linear Stern-Volmer plot is generally an indicative of a homogeneous population of emitting fluorophores [24], i.e., a single class of fluorophore, all equally accessible to the quencher, which indicates that only one mechanism (dynamic or static) of quenching occurs [22].

However, in many cases, positive deviations from the Stern-Volmer equation occurred suggesting that the fluorophore population is heterogeneous [24]. Namely, the fluorophore is quenched by both mechanisms (dynamic and static) simultaneously or the presence of a sphere of action [24]. As a result, the Stern-Volmer equation should be modified to Eq. 4:
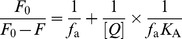
(4)where: *f*_a_ is the fraction of fluorophore accessible to the quencher, and *K*_A_ is the modified Stern-Volmer quenching constant, which is very close to the binding constant. *f*_a_ and *K*_A_ values can be calculated from the intercept and slope of the *F*_0_ */(F*_0_ *–F*) vs. 1/[Q] plot.

### 5. DLS analysis for the measurement of hydrodynamic radius (R_h_)

Zetasizer Nano series of DLS (Malvern Instruments Ltd., Worcestershire, UK) was employed to measure the hydrodynamic radii of PL. PL (2.5 µmol/L) and EGCG analogs (0–1 mmol/L) were mixed and incubated at 37°C for 45 min before determination. The path length of the cuvette was 10 mm. Triplicate measurements were done and the average value of hydrodynamic radius was presented [19], [25].

### 6. Kinetic parameters (K_m_ and V_max_) assessment

In order to measure the kinetic parameters *K*_m_ and *V*_max_ in the Michaelis-Menten equation, the effect of substrate concentration on the initial reaction velocity was studied through the hydrolysis reaction by PL using olive oil as a substrate. The PL solutions (2.5 µmol/L) in the absence or presence of EGCG analogs (0.25 and 0.5 mmol/L) were incubated with various concentrations of emulsified olive oil with the final concentrations of 0.03, 0.06, 0.08, 0.09, and 0.10 mg/mL, respectively. In all cases, the enzymatic activity in phosphate buffer (pH 7.4) was assayed at 37°C and detailed in section 2.2 [18]. From the Lineweaver–Burk plots, the Michaelis constant (*K*_m_) and maximum velocity (*V*_max_) can be determined [26].

### 7. Thermodynamic parameter (T_m_) assessment with DSC

Thermodynamic parameter *T_m_*, the transition midpoint temperature, was determined by a VP-DSC (MicroCal, Northampton, MA). Temperature scans were from 30 to 70°C at a scan rate of 1°C/min. PL (2.5 µmol/L) solution in the absence or presence of a certain EGCG analogs (0–1 mmol/L) was incubated at 37°C for 45 min before analysis. The sample cell was loaded with the PL solution and the same concentration phosphate buffer was loaded into the reference cell as the blank control. The samples and control were degassed for 15 min immediately before DSC scanning. A buffer-buffer reference scan was subtracted from each sample scan prior to concentration normalization. DSC data were analyzed by MicroCal Origin Version 7.0.

## Results and Discussion

### 1. Effects of EGCG analogs on PL activity

**Figure 2:**
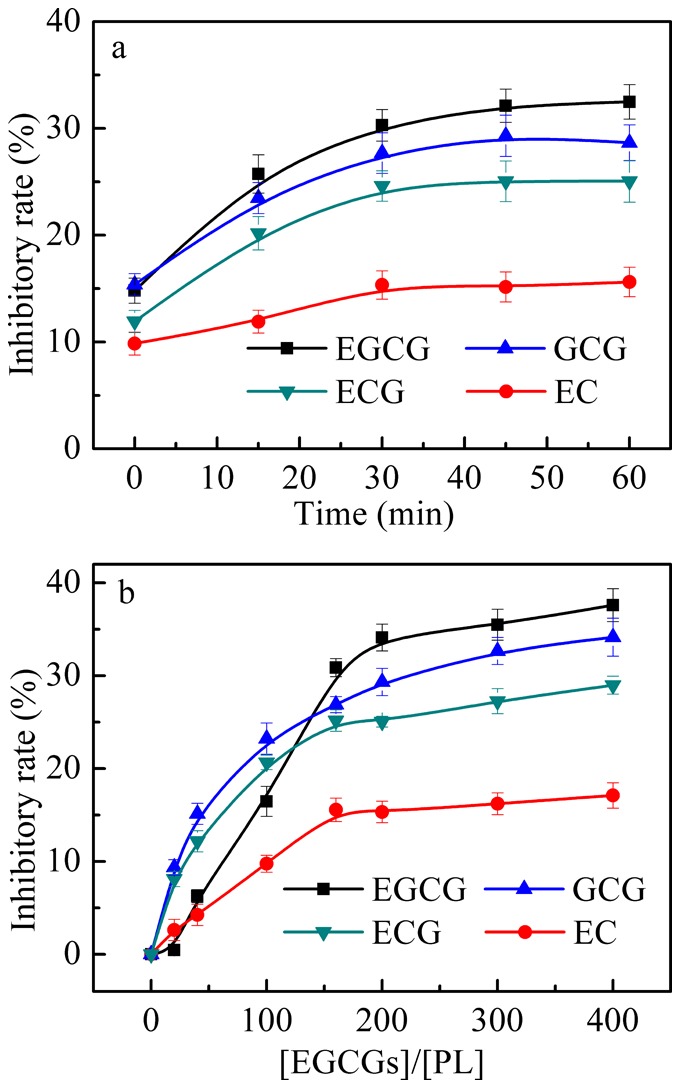
Effects of incubation time (a) and EGCG analogs concentration (b) on the PL activity. The concentration of PL was 2.5 µmol/L. The concentrations of EGCG analogs in Figure 2a were 0.50 mmol/L. The concentrations of EGCG analogs in Figure 2b can be calculated from the [EGCG analogs]/[PL] ratio, which were in the range of 0–1 mmol/L. In Figure 2b, the activity was measured after a 45-min incubation of the mixture of PL and EGCG analogs at 37°C. All trials were carried out in 100 mmol/L PBS (pH 7.4) at 37°C.

The effects of incubation time on the activity of PL were investigated and the results are depicted in Figure 2a. It was found that the activity of PL alone was independent of time within 60 min incubation. With EGCG analogs, the activity decreased in the order of EC>CG>GCG>EGCG. The activity gradually decreased with time and reached almost constant at 45 min, indicating that the binding of EGCG analogs to PL reached equilibrium at this time (45 min). Hence, the following experiments were all conducted after 45 min incubation.

The influences of EGCG analogs concentration (*r* value) on the hydrolysis activity of PL were evaluated and the results were presented in Figure 2b. It was observed that the overall changing trends of the PL activity with the four analogs as a function of *r* value were similar to each other. Namely, the activity first decreased rapidly with increasing *r* value at *r*<*ca*. 180, and then decreased slowly with the increase of *r* value at 180<r<400. However, at *r*<180, the profiles for EGCG and EC were almost linear, while those for GCG and ECG were quadratic. Although the activity for EGCG was higher than those of GCG and ECG at *r*<110–220, it was lower than the two molecules at *r*>*ca*. 220.

### 2. Effects of EGCG analogs on PL secondary structure

**Figure 3:**
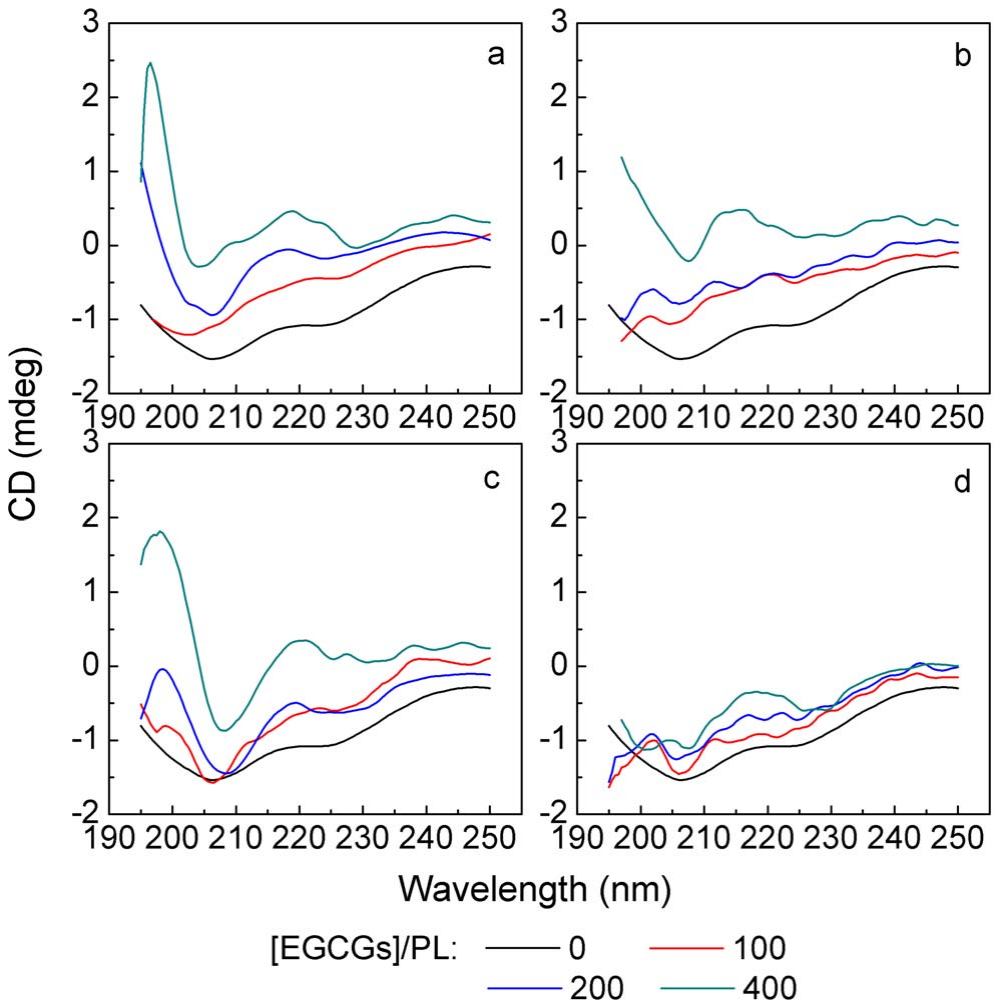
Effects of EGCG analogs on the far-UV CD spectra of PL. a–d: EGCG, GCG, ECG, and EC. The final concentration of PL was 1 µmol/L. The concentrations of EGCG analogs can be calculated from the [EGCG analogs]/[PL] ratio, which were in the range of 0–0.4 mmol/L. Measurements were done after a 45-min incubation of the mixture of PL and EGCG analogs at 37°C. Experiments were carried out in 100 mmol/L PBS (pH 7.4).

The effects of EGCG analogs on the secondary structure of PL were evaluated. The CD spectra are provided in Figure 3 and the percentages of the protein secondary structural elements derived from the spectra are listed in Table 1.

**Table 1:** Effects of EGCG analogs on the contents of secondary structure of PL.

EGCG analogs	*r* = [EGCG analogs]/[PL] *	Helix (%)	Sheet (%)	Turn (%)	Unordered (%)
PL	0	13.7	32.5	20.5	33.3
EGCG	100	11.6	36.7	21.9	29.8
	200	9.1	37.1	22.4	31.4
	400	7.2	39.6	21.1	32.1
GCG	100	11.8	35.4	22.1	30.7
	200	9.9	36.1	22.2	31.8
	400	7.8	38.9	21.8	31.5
ECG	100	12.1	33.5	22.3	32.1
	200	10.9	35.1	22.7	31.3
	400	8.8	37.5	22.8	30.9
EC	100	13.2	33.1	21.1	32.6
	200	12.4	34.2	21.6	31.8
	400	10.7	36.5	22.4	30.4

*The concentration of PL was 1 µmol/L. The concentrations of EGCG analogs can be calculated from the [EGCG analogs]/[PL] ratio, which were in the range of 0–0.4 mmol/L. Experiments were carried out in 100 mmol/L PBS (pH 7.4).

As shown in Figure 3, the CD spectrum of PL alone showed both minimum at 208 and 222 nm, indicating that the enzyme possessed α-helix. By further fitting the spectrum, it is showed that PL contained 13.7% α-helix and 32.5% β-sheet (see Table 1). The addition of EGCG analogs concentration dependently decreased the signal at 208 and 222 nm, which indicated that EGCG analogs changed the secondary structure of PL to different extents. For example, as listed in Table 1, EGCG decreased the α-helix of PL from 13.7% to 7.2% and increased the β-sheet from 32.5% to 39.6% with the increase of [EGCG]/[PL] ratio from 0 to 400. GCG ECG and EC showed similar influences to the secondary structure of PL, and the changing extents of the four EGCG analogs increased in the order of EC<ECG<GCG<EGCG (Figure 3 and Table 1).

By comparing with Figure 2, it is seen that the decrease of α-helix was related to the reduction of PL activity. This phenomenon has been observed in many previous studies [18], [19]. As well known, PL contains 449 of amino acid residues. The N-terminal domain of it comprises residues 1–335 and is typical of α/β structure. The C-terminal domain is of the β-sandwich type [27]. Around the catalytic site, there are several α-helices which stabilize the enzyme and maintain its activity [27], [28]. Therefore, the decrease of α-helix can destabilize the enzyme and reduce its activity.

### 3. Effects of EGCG analogs on PL Fluorescence spectra

**Figure 4:**
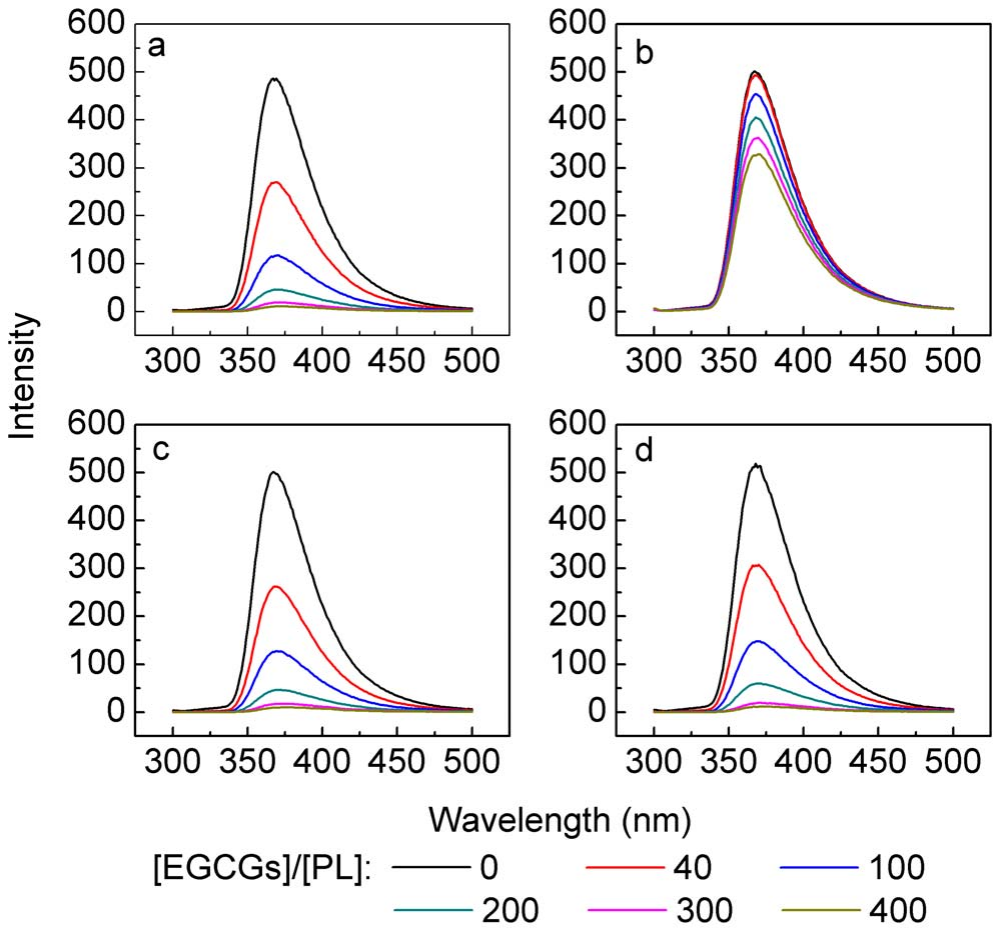
Effects of EGCG analogs on the fluorescence spectra of PL. a–d: EGCG, GCG, ECG, and EC. The concentration of PL was 2.5 µmol/L. The concentrations of EGCG analogs can be calculated from the [EGCG analogs]/[PL] ratio, which were in the range of 0–1 mmol/L. Measurements were done after a 45-min incubation of the mixture of PL and EGCG analogs at 37°C. Experiments were carried out in 100 mmol/L PBS (pH 7.4).

The intrinsic fluorescence spectra of PL in the absence or presence of EGCG analogs are compared in Figure 4. Generally, the fluorescence emission maximum values of the four EGCG analogs were independent of *r* values, indicating that the microenvironment of tryptophan in PL was not changed by EGCG analogs [22].

The fluorescence intensity of the four molecules decreased with the increase of *r* value, suggesting that the fluorescence of PL was quenched by EGCG analogs. Fluorescence quenching is generally classified into two types: dynamic quenching, resulting from collisional encounters between the fluorophore and the quencher, and static quenching, resulting from the formation of a ground state complex between the fluorophore and the quencher [29], [30]. If only one type of quenching occurs (either dynamic or static), it can be described by the linear Stern-Volmer equation (Eq. 3) [22]. However, in most cases, the two types of quenching occur simultaneously. As a result, the *F*_0_/*F* vs. [*Q*] plot in Eq. 3 exhibits an upward curvature. In this situation, the Stern-Volmer equation will be modified to Eq. 4 to obtain a linear relationship.

In our work, PL protein suffered both static and dynamic quenching upon the addition of EGCG, ECG, and EC, so the modified Stern-Volmer equation (Eq. 4) was utilized, and the parameters *f*_a_ and *K*_A_ were obtained from the slope and intercept of the equation. As listed in Table 2, the *K*_A_ value standing for the binding constant decreased by the order of EGCG>ECG>EC, which was in consistence with the PL activity in Figure 2, i.e., the higher *K*_A_ value, the lower the activity of PL. Additionally, the *K*_A_ values of proanthocyanidins (PC) and PL reported in our previous work was 12.3±0.4×10^3^ L/mol (Table 2 therein) [19], higher than those of EGCG, ECG, and EC. It suggested that the binding affinity of the three molecules to PL was lower than that of PC.

**Table 2:** Modified Stern-Volmer constant (*K*_A_) and the fraction of fluorophore accessible to the quencher (*f*_a_) for the interactions of EGCG analogs with PL.

EGCG analogs	*K*_A_ (L/mol)	*f*_a_
EGCG	8820±90	0.87
ECG	8240±50	0.99
EC	7550±80	0.81

Four of six Trp residues in PL structure were fully buried (Trp86, Trp106, Trp252, and Trp402). The other two were exposed to solvent (Trp 117, Trp 338) with the exposure percentage lower than 9% [23]. Parameter *f*_a_ stands for the fraction of fluorophore accessible to the quencher. Since the *f_a_* values listed in Table 1 were much higher than the exposed percentage of Trp (about 9%), it could be rationally speculated that EGCG analogs quenched both the external and internal Trp fluorescence of PL [22], [30]. In addition, the *f*_a_ value of PC to PL was about 0.88 [19], which was similar to those listed in Table 2, indicating that the fraction of fluorophore accessible to the quencher was similar for EGCG, ECG, EC and PC.

However, in view of GCG (Figure 4b), the single type of quenching occurred because the Stern-Volmer equation fitted well. The parameters *K*_SV_ and 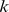
_q_ were calculated to be 740±15 L/mol and 4.65×10^11^ L/mol/s. Since the bimolecular quenching constant 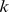
_q_ was higher than 10^10^ L/mol/s, the possible maximum value for diffusion-limited quenching (dynamic mechanism) in aqueous medium, it could be concluded that the fluorescence quenching was static with a formation of GCG-PL complexes [30]. Skrt et al. studied the interactions between bovine serum albumin (BSA) and EGCG, ECG, EC, and they reported that the *K*_SV_ values between BSA and the three tea polyphenols are 9.22±0.03×10^5^, 1.361±0.013×10^6^, and 4.92±0.02×10^4^L/mol, respectively. The 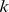
_q_ values between them were 184±0.6×10^12^, 272±3×10^12^, and 9.8±0.1×10^12^ L/mol/s, respectively [31]. In our previous work, the *K*_SV_ and 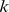
_q_ values between PL and PC were 2.01±0.04×10^4^ and 1.26±0.05×10^13^ [19]. By comparing these results, it could be seen that the molecular interactions between GCG and PL were relatively weak.

It should be noted that although the four analogues all could form complexes with PL due to the occurrence of static quenching, the fluorescence emission maximum values kept the same (Figure 4). It inferred that only weak molecular interactions were involved in the binding affinity of EGCG analogs to PL in this work.

### 4. Effects of EGCG analogs on the hydrodynamic radius (R_h_) of PL

**Figure 5:**
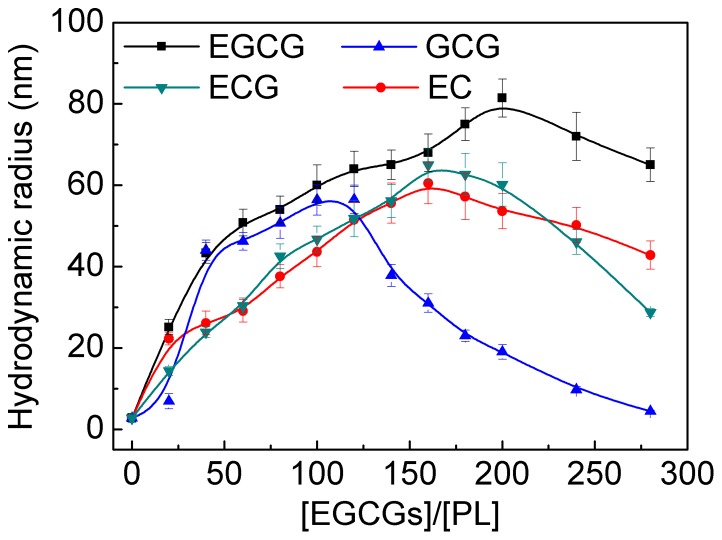
Effects of EGCG analogs on the hydrodynamic radius (*R*_h_) of PL. The concentration of PL was 2.5 µmol/L. The concentrations of EGCG analogs can be calculated from the [EGCG analogs]/[PL] ratio, which were in the range of 0–0.75 mmol/L. Measurements were done after a 45-min incubation of the mixture of PL and EGCG analogs at 37°C. Experiments were carried out in 100 mmol/L PBS (pH 7.4).

Figure 5 shows the influences of EGCG analogs on the hydrodynamic radius (*R*_h_) of PL. With the increase of EGCG analogs concentration, the *R*_h_ values all presented bell-shaped profiles. Namely, the *R*_h_ values first increased with the increase of *r* value, reached the peak at a certain *r*, then decreased with further increase of *r* value. From the *R*_h_ values, it was concluded that PL proteins self-assembled into soluble complexes through the link of EGCG analogs. The results were in highly agreement with those reported between EGCG and insulin [25]. Insulin assembled into complexes through the link of EGCG, and the radius curve of the complex as a function of EGCG concentration was bell-shaped [25]. However, the inhibitory mechanisms of EGCG analogs to PL was significantly different from that of PC to PL, because the profile of *R*_h_ value of PC-PL complex as a function of PC concentration was sigmoidal [19].

The turning points of the bell-shaped curves for EGCG, ECG, and EC in Figure 5 were at *R*_h_ values of 200, 160, and 160, respectively. Combining with the data in Figure 2b, it could be reasonably speculated that the *R*_h_ value was closely related to the PL activity. Before the turning point, the activity rapidly decreased with the increase of *R*_h_ value. After turning point, although the *R*_h_ value of the complex decreased, the concentrations of EGCG analogs still increased, therefore the reverse effects of the two factors finally resulted in the slow decrease of the activity.

However, in view of GCG-PL interaction, the turning point of *R*_h_ was 120, while the turning point of PL activity was 160 (Figure 2b). The reason might be explained from the different quenching mechanisms for different EGCG analogs. EGCG, ECG, and EC quenched the Trp fluorescence of PL both dynamically and statically. It indicated that part of the molecules formed complexes with PL and the others just encountered PL collisionally. On the contrary, GCG only quenched the Trp fluorescence statically, i.e., all the molecules was bound with PL to form complexes, so the effective concentration of GCG was higher than those of the other three molecules. Therefore, the turning point of GCG was apparently lower than the turning point of PL activity.

### 5. Effects of EGCG analogs on the kinetics parameters (K_m_ and V_max_) of PL

**Figure 6:**
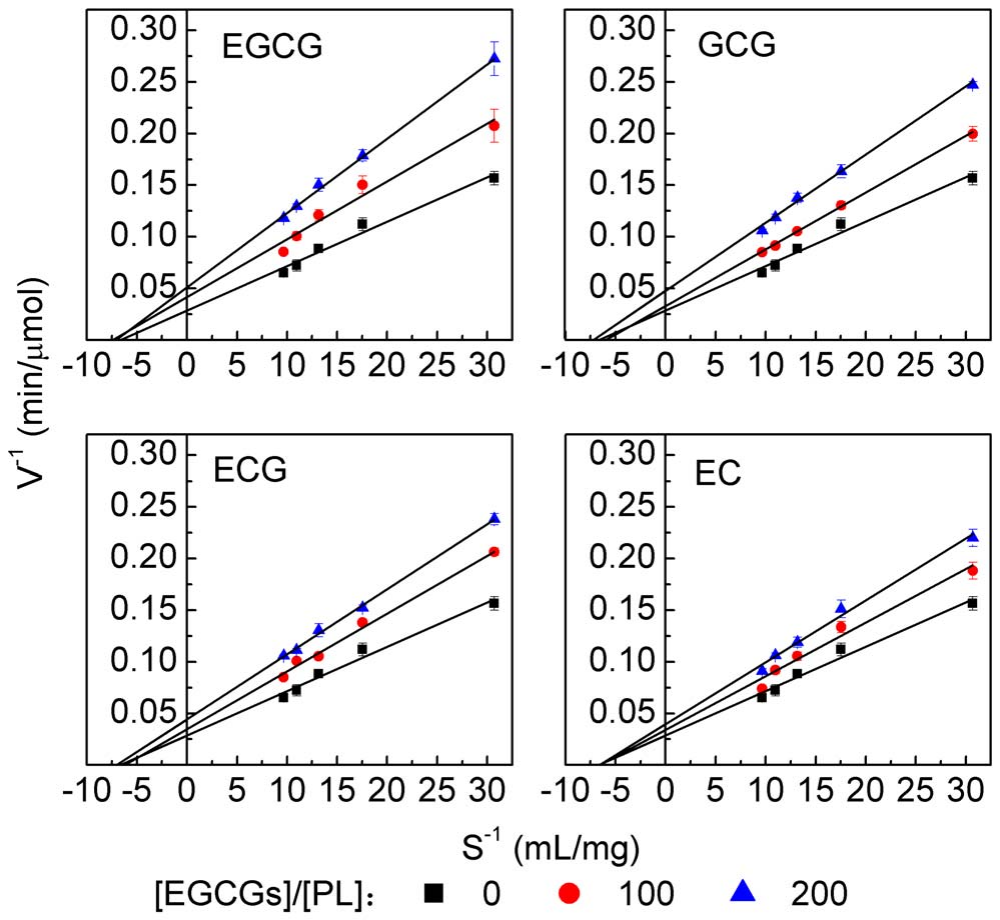
Lineweaver-Burk plot for the PL activity at different EGCG analogs concentrations. The concentration of PL was 2.5 µmol/L. The concentrations of EGCG analogs can be calculated from the [EGCG analogs]/[PL] ratio, which were in the range of 0–0.5 mmol/L. Measurements were done after a 45-min incubation of the mixture of PL and EGCG analogs at 37°C. Experiments were carried out in 100 mmol/L PBS (pH 7.4).

To investigate the influences of EGCG analogs on the Michaelis-Menten constant (*K*_m_) and the maximum velocity (*V*_max_) of PL, Lineweaver-Burk equation is plotted in Figure 6 and the *K*_m_ and *V*_max_ values derived from the slope and intercept of the equation are listed in Table 3. From the table, it was observed that the *K*_m_ and *V*_max_ of PL alone (i.e., [EGCG analogs]/[PL] = 0) were 0.15±0.01 mg/mL and 34.97±2.21 µmol/min, respectively. The addition of EGCG analogs almost did not change the *K*_m_ value of PL, but decreased the *V*_max_ value in a concentration dependent manner. The results inferred that EGCG analogs non-competitively inhibited the PL activity and would not bind to the catalytical site of the enzyme. The conclusion agreed well with the above results that many EGCG analogs molecules were bound to PL and induced PL assemble into complexes. The complex prevented the access of the substrate by spatial hindrance and reduced the affinity between the enzyme and its hydrophobic substrate (olive oil).

**Table 3:** Effect of EGCG analogs concentration on the kinetic parameters of PL (*K*_m_ and *V*_max_).

EGCG analogs	*r* = [EGCG analogs]/[PL] *	*K*_m_ (mg/mL)	*V*_max_ (µmol/min)	*V*_max_/*K*_m_
PL	0	0.15±0.01	34.97±2.21	232
EGCG	100	0.13±0.01	24.04±1.93	178
	200	0.14±0.02	19.61±1.66	138
GCG	100	0.16±0.02	26.49±2.02	165
	200	0.14±0.01	21.01±1.98	152
ECG	100	0.16±0.02	28.90±2.35	179
	200	0.14±0.01	22.62±1.69	159
EC	100	0.15±0.01	29.41±2.33	192
	200	0.15±0.01	25.32±2.45	167

*The concentration of PL was 2.5 µmol/L. The concentrations of EGCG analogs can be calculated from the [EGCG analogs]/[PL] ratio, which were in the range of 0–0.5 mmol/L. Experiments were carried out in 100 mmol/L PBS (pH 7.4) at 37°C.

The data in Table 3 also showed that the *V*_max_ and *V*_max_/*K*_m_ values standing for the enzymatic catalytic intensity increased by the order of EGCG>GCG>ECG>EC, which was positively related to the PL activity (Figure 2b). The decrease of *V*_max_/*K*_m_ with the increase of *r* value indicated that EGCG analogs not only reduced the reaction initial rate [18], but also weakened the enzymatic catalytic intensity [26].

### 6. Effects of EGCG analogs on the thermodynamic parameter (T_m_) of PL

**Figure 7:**
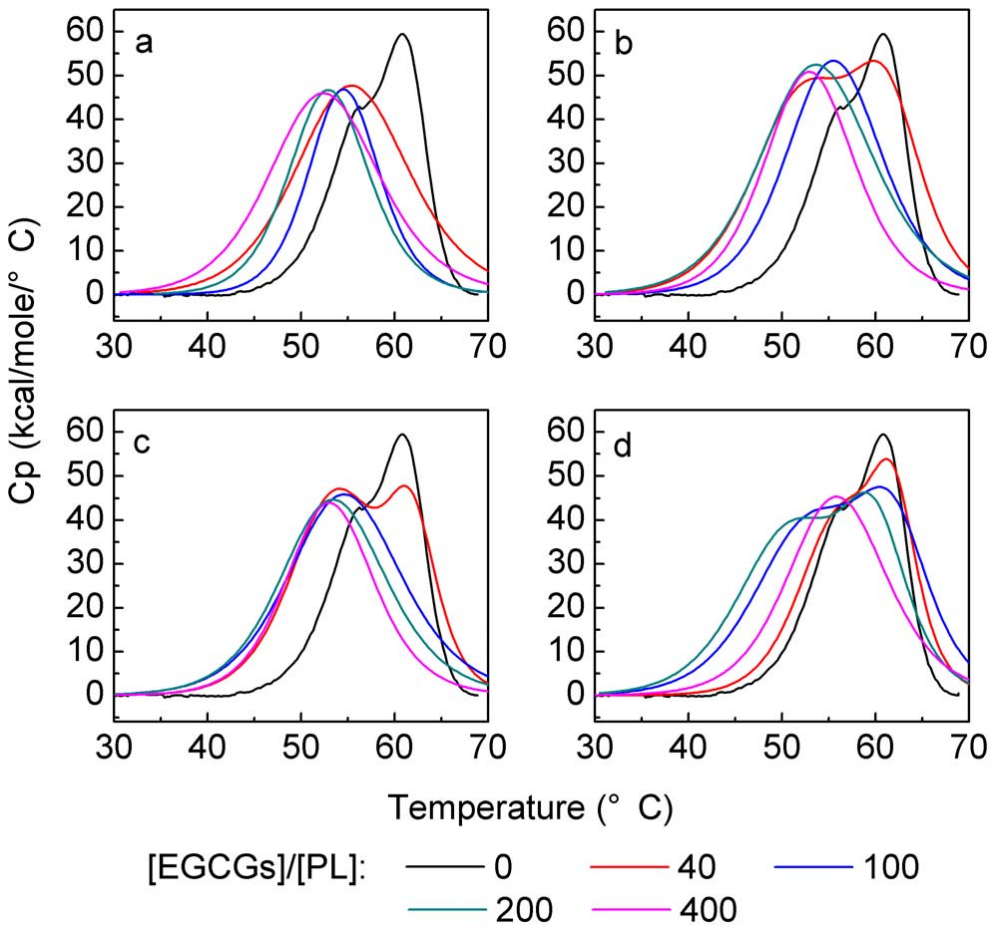
Effects of EGCG analogs on the DSC curve of PL. a–d: EGCG, GCG, ECG, and EC. The concentration of PL was 2.5 µmol/L. The concentrations of EGCG analogs can be calculated from the [EGCG analogs]/[PL] ratio, which were in the range of 0–1 mmol/L. Measurements were done after a 45-min incubation of the mixture of PL and EGCG analogs at 37°C. Experiments were carried out in 100 mmol/L PBS (pH 7.4).

The effects of EGCG analogs on the thermostability of PL were evaluated. The DSC curves of PL in the absence or presence of EGCG analogs at different *r* values are shown in Figure 7 and the transition midpoint temperature (*T*_m_) values picked from the curves are listed in Table 4.

**Table 4:** Effects of EGCG analogs concentrations on the transition midpoint temperature (*T*_m_) of PL.

EGCG analogs	*r* = [EGCG analogs]/[PL]*	*T*_m1_ (°C)	*T*_m2_ (°C)
PL	0	56.4±0.3	61.5±0.2
EGCG	40	55.3±0.4	
	100	54.4±0.5	
	200	52.8±0.6	
	400	52.2±0.5	
GCG	40	52.7±0.8	60.0±0.4
	100	55.3±0.5	
	200	53.4±0.5	
	400	52.8±0.4	
ECG	40	53.0±0.3	61.1±0.4
	100	54.5±0.3	
	200	53.3±0.3	
	400	52.9±0.3	
EC	40	56.1±0.2	61.9±0.2
	100	52.2±0.3	61.5±0.4
	200	50.6±0.2	58.7±0.3
	400	55.7±0.2	

*The concentration of PL was 2.5 µmol/L. The concentrations of EGCG analogs can be calculated from the [EGCG analogs]/[PL] ratio, which were in the range of 0–1 mmol/L. Experiments were carried out in 100 mmol/L PBS (pH 7.4).

The data in Table 4 showed that PL alone possessed two transition midpoint temperatures with the values of 56.4 and 61.5°C, respectively. The results were similar to those of *Rhizopus niveus* lipase whose *T*_m_ values were 55.2 and 62.1°C reported by Rabbani et al. [32]. The reason why lipase displayed two *T*_m_ values was that lipase has two functionally important sites, a catalytic site and a topographically distinct interfacial ‘recognition site’ [27]. Upon the addition of EGCG, the *T*_m2_ value of PL disappeared because the binding of EGCG to PL would change the functionally important sites of enzyme. Since EGCG non-competitively inhibited the PL activity, i.e., EGCG could not bind to the catalytic site, it was inferred that EGCG changed the structure of the topographically distinct interfacial ‘recognition site’, which prevented the access of the substrate and reduced the affinity between the enzyme and the substrate. On the other hand, EGCG just reduced the *T*_m1_ value to some extent. The results agreed with the CD results that EGCG might disturb the α-helices around the catalytic site and therefore destablize the catalytic site to some degree. However, PL still remained about 60% of its original activity at *r* = 400 (see Figure 2b).

As for GCG and ECG, at *r* = 40, the *T*_m1_ and *T*_m2_ values of PL were both decreased (Table 4). At *r*>100, the condition was similar to that of EGCG. Namely, the *T*_m2_ value disappeared and the *T*_m1_ value decreased with increasing *r*. In addition, the *T*_m1_ values at *r*>100 decreased in the order of ECG>GCG>EGCG, and the PL activity decreased in the same order of ECG>GCG>EGCG (Figure 2b).

As for EC, the *T*_m1_ and *T*_m2_ values of PL at *r*<200 were decreased dependently on EC concentration. At *r* = 400, EC greatly changed the recognition site of the enzyme and therefore *T*_m2_ disappeared. However, the change of PL's structure imposed by EC was weaker than those of EGCG, GCG, and ECG due to the higher *T*_m2_ value, so the inhibitory effect of EC was much lower than the other three analogues molecules (Figure 2a and b). Different from EGCG analogs, PC could increase the thermostability of PL although these compounds are of polyphenols. It was demonstrated that, at *r*<40, PC had little effect on *T*_m1_ and *T*_m2_ values of PL. At *r*>50, both *T*_m1_ and *T*_m2_ disappeared and another peak with a *T*_m_ value of 76.9 appeared [19]. Therefore, different type of polyphenols will show different inhibitory mechanism of PL in view of the thermostability.

### 7. Molecular interaction mechanism between PL and EGCG analogs

In this study, molecular interactions between EGCG analogs and PL were investigated from various aspects to compare the differences between the four analogues and analyzed the contributions of galloyl moiety and 3′-hydroxyl group in EGCG analogs structure on the inhibition rate of PL. Results showed that EGCG analogs inhibited the activity of PL. The PL activity first decreased rapidly with increasing *r* value at *r*<ca. 180, and then decreased slowly with the increase of *r* value at 180<r<400 (Figure 2b). EGCG analogs induced the self-assembly of PL into complexes and the *R*_h_ value first increased and then decreased with the increase of *r* value (Figure 5). Since the turning points of the PL activity were closely related to the turning points of *R*_h_, it could be reasonably speculated that the formation of complex suppressed the access of substrates to the enzyme and inhibited the PL activity. On the other hand, it could also be inferred from the non-competitive inhibition that EGCG analogs might not bind to the catalytic site of PL (Figure 6 and Table 3). In combination with the analyses of CD and DSC (Figures 3 and 7, Tables 1 and 4), it could be speculated that EGCG analogs changed the recognition site of PL, which prevented the substrate from access to PL (section 3.6), and to some extent altered the catalytic site of PL. Therefore, it reduced the activity of PL (sections 3.2 and 3.6).

FS results revealed that EGCG analogs formed complexes with PL due to the occurrence of static quenching, while the fluorescence emission maxima were unchanged. From the results, it can be inferred that the interactions between EGCG analogs and PL were weak and nonspecific. To further confirm this conclusion, we added 2 mol/L urea solutions to the PL-EGCG mixture since low concentration of urea solution was known to destroy weak and non-covalent interactions between protein and small molecules. As shown in Figure S2 in File S1, the activity of PL was recovered after the addition of urea solution. Comparing Figure S3 in File S1 with Figure 3, the secondary structure of PL was almost recovered by urea solution. Moreover, we subjected the PL-EGCG mixture to SDS-PAGE to test whether the PL-EGCG mixture can be disassembled by sodium dodecyl sulfonate (SDS), which is often used to destroy non-covalent interactions and denature proteins. As shown in Figure S4 in File S1, only PL monomer was presented in each panel, indicating that the PL-EGCG complex can be fully disassembled by SDS. The results for GCG, ECG, and EC were similar to those for EGCG (data not shown). These findings indicated that the interactions between EGCG analogs and PL were weak and non-covalent, and the bindings were reversible. The conclusions were in agreement with many reports on that EGCG bound with other proteins, e.g., amyloid beta (Aβ) protein, α-synuclein, insulin, islet amyloid polypeptide, α-caseins, β-caseins, through non-covalent and non-specific interactions [33]–[37]. Furthermore, through analyses of FT-IR spectroscopy, isothermal titration calorimetry, and dynamic light scattering, the weak interactions involved in the binding of polyphenols to proteins were confirmed to be mainly hydrogen bonding and hydrophobic interaction [21], [37], [38].

Among the four tea polyphenols, EGCG showed the best inhibitory effect to PL activity, followed by GCG and ECG, and EC possessed the least effect (Figure 2). As provided in Figure 1, the structural difference between EGCG and ECG lies on the 3′-hydroxyl group. It indicated that the 3′-hydroxyl group might be crucial to the inhibition of PL. This phenomenon agreed well with those reported in literature [39]. Although hydroxyl group can increase the binding affinity to PL through hydrogen bonding, the loss of 3′-hydroxyl group cannot lead to the change of quenching mechanism and inhibitory type (Table 2 and Figure 6).

The structural difference between EGCG and EC is mainly the galloyl moiety (the D ring as shown in Figure 1), and the inhibitory rate of EC on PL was 20.5% lower than that of EGCG (calculated from Eq. 2 and Figure 2). The galloyl moiety contains one phenyl group and three hydroxyl groups, so it binds to PL mainly through hydrophobic interactions and hydrogen bonding. The loss of galloyl moiety would greatly lower the binding affinity to PL, so EC showed slightly effects on the conformation and thermostability of PL (Figures 3 and 4, Tables 1 and 4). Simultaneously, EC did not change the quenching mechanism and inhibitory type to PL (Table 2 and Figure 6). However, the *R*_h_ values of ECG-PL and EC-PL complexes were almost similar (Figure 5). This result was probably explained by two factors. On the one hand, the loss of galloyl moiety reduced the binding affinity to PL, which might lead to the decrease of *R*_h_; on the other hand, the loss of galloyl moiety decreased the steric hindrance, which would facilitate the formation of complexes. Consequently, galloyl moiety almost presented little effect on the *R*_h_ value. Ishii et al. [40] compared the interactions between human serum albumin (HSA) and EGCG as well as EC. The authors pointed out that the galloyl moiety participated in the interaction of EGCG with HAS, and the interaction was of critical importance in preventing EGCG oxidation.

GCG is an epimer of EGCG, and the structural difference between them lies on B ring (Figure 1). As can be seen in Figure 2b, the difference of inhibitory rate between EGCG and GCG could be neglected (*ca.* 3.5% calculated from Eq. 2). The small difference indicated that the interactions between EGCG analogs and PL were non-specific interactions. It was obviously observed that the influence of EGCG on the PL conformation and thermostability as well as the inhibitory type was similar to the effect of GCG (Figures 3, 4, and 6, Tables 1, 3, and 4). However, owing to the structural difference of EGCG and GCG, the quenching mechanisms of them to PL showed discrepancy. Specifically, EGCG quenched the fluorescence of PL both dynamically and statically, which indicated that part of the EGCG molecules formed complexes with PL and some just encountered PL collisionally; while GCG only quenched the fluorescence statically, suggesting that all the molecules bound to PL to form complexes (section 3.3). The difference of quenching mechanism directly led to the different binding constant (EGCG, 8820±90 L/mol in Table 2; GCG, 740±15 L/mol) and profiles of *R*_h_ values (Figure 5) of EGCG and GCG.

## Conclusions

In this study, the molecular interactions between PL and four EGCG analogs, i.e., EGCG, GCG, ECG, and EC were investigated by CD, FS, DLS, and DSC approaches. Results showed that the EGCG analogs showed different effect on activity, conformation, thermodynamics and kinetics of PL. Structural units of 3′-hydroxyl group, galloyl moiety (D ring), and B ring in EGCG analogs structure were probably the key factors. The findings of our work could shed insights on the inhibitory mechanisms of EGCG analogs to PL, and contribute to the development of the natural effective inhibitors for the prevention of human obesity in future.

## Supporting Information

File S1 **Figures S1–S4 and Table S1.** Figure S1. RP-HPLC spectra of EGCG incubated for 0 or 2 h. EGCG of 1 mmol/L was dissolved in 100 mmol/L PBS (pH 7.4) at 37°C. EGCG was analyzed on an Agilent 1100 HPLC system (Agilent Technologies, Palo Alto, CA, USA) using the ZORBOX 300-SB C18 column. Eluant A (5% aqueous methanol with 0.2% acetic acid) and eluant B (95% aqueous methanol with 0.2% acetic acid) were used for this analysis. The elution was achieved by a linear gradient of 0–100% eluant B mixed with eluant A in 50 min at 1.0 mL/min. The column effluent was monitored at 280 nm. Figure S2. Activity of urea-treated PL and EGCG complexes. The concentrations of PL and EGCG were 5 µmol/L and 0.5–2 mmol/L, respectively, in the original solutions. The solutions were incubated in 100 mmol/L PBS (pH 7.4) at 37°C for 45 min. After that, urea solution was added, and the final concentrations of urea, PL, and EGCG were 2 mol/L, 2.5 µmol/L and 0.25–1 mmol/L, respectively. PL activity was measured after dialysis. In the figure, the number 100, 200, 300, and 400 denoted the [EGCG]/[PL] ratio and the capital U meant the mixture was treated by urea solution. The activities of PL alone (PL), PL with urea solution (PL+U), and PL-EGCG mixture without urea (number 100–400) were compared. Figure S3. Secondary structures of urea-treated PL and EGCG complexes. a, CD spectra of urea-treated PL and EGCG complexes. The concentrations of PL and EGCG were 5 µmol/L and 0.5–2 mmol/L, respectively, in the original solutions. The solutions were incubated in 100 mmol/L PBS (pH 7.4) at 37°C for 45 min. After that, urea solution was added, and the final concentrations of urea, PL, and EGCG were 2 mol/L, 2.5 µmol/L and 0.25–1 mmol/L, respectively. Before measurement, solutions were diluted 2.5-folds to meet the limit of detection. b, the contents of secondary structure of urea-treated PL and EGCG complexes. Figure S4. SDS-PAGE of PL-EGCG mixture. The concentrations of PL and EGCG were 2.5 µmol/L and 0.25–1 mmol/L, respectively. The solutions were incubated in 100 mmol/L PBS (pH 7.4) at 37°C for 45 min before measurement. The [EGCG]/[PL] ratios from panel 1 to 4 were 0, 100, 200, and 400, respectively. Table S1. Absorbance of PL-EGCG mixture at 500 nm (A500). The concentrations of PL and EGCG were 2.5 µmol/L and 0.25–1 mmol/L, respectively. Experiments were done in 100 mmol/L PBS (pH 7.4) at 37°C. The A500 of EGCG alone (1 mmol/L) and PL alone were compared.
(DOC)
