# Supplementary material for: Molecular Interactions between (−)-Epigallocatechin Gallate Analogs and Pancreatic Lipase
Source: PLoS One. 2014 Nov 3;9(11):e111143. doi: 10.1371/journal.pone.0111143 (PMC4218840; doi:10.1371/journal.pone.0111143)
Supplement: File S1 — Figures S1–S4 and Table S1. Figure S1. RP-HPLC spectra of EGCG incubated for 0 or 2 h. EGCG of 1 mmol/L was dissolved in 100 mmol/L PBS (pH 7.4) at 37°C. EGCG was analyzed on an Agilent 1100 HPLC system (Agilent Technologies, Palo Alto, CA, USA) using the ZORBOX 300-SB C18 column. Eluant A (5% aqueous methanol with 0.2% acetic acid) and eluant B (95% aqueous methanol with 0.2% acetic acid) were used for this analysis. The elution was achieved by a linear gradient of 0–100% eluant B mixed with eluant A in 50 min at 1.0 mL/min. The column effluent was monitored at 280 nm. Figure S2. Activity of urea-treated PL and EGCG complexes. The concentrations of PL and EGCG were 5 µmol/L and 0.5–2 mmol/L, respectively, in the original solutions. The solutions were incubated in 100 mmol/L PBS (pH 7.4) at 37°C for 45 min. After that, urea solution was added, and the final concentrations of urea, PL, and EGCG were 2 mol/L, 2.5 µmol/L and 0.25–1 mmol/L, respectively. PL activity was measured after dialysis. In the figure, the number 100, 200, 300, and 400 denoted the [EGCG]/[PL] ratio and the capital U meant the mixture was treated by urea solution. The activities of PL alone (PL), PL with urea solution (PL+U), and PL-EGCG mixture without urea (number 100–400) were compared. Figure S3. Secondary structures of urea-treated PL and EGCG complexes. a, CD spectra of urea-treated PL and EGCG complexes. The concentrations of PL and EGCG were 5 µmol/L and 0.5–2 mmol/L, respectively, in the original solutions. The solutions were incubated in 100 mmol/L PBS (pH 7.4) at 37°C for 45 min. After that, urea solution was added, and the final concentrations of urea, PL, and EGCG were 2 mol/L, 2.5 µmol/L and 0.25–1 mmol/L, respectively. Before measurement, solutions were diluted 2.5-folds to meet the limit of detection. b, the contents of secondary structure of urea-treated PL and EGCG complexes. Figure S4. SDS-PAGE of PL-EGCG mixture. The concentrations of PL and EGCG were 2.5 µmol/L and 0.25–1 mmol [file pone.0111143.s001.doc]

**Supporting Information Legends**

Figure S1. RP-HPLC spectra of EGCG incubated for 0 or 2 h. EGCG of 1 mmol/L was dissolved in 100 mmol/L PBS (pH7.4) at 37 °C. EGCG was analyzed on an Agilent 1100 HPLC system (Agilent Technologies, Palo Alto, CA, USA) using the ZORBOX 300-SB C18 column. Eluant A (5% aqueous methanol with 0.2% acetic acid) and eluant B (95% aqueous methanol with 0.2% acetic acid) were used for this analysis. The elution was achieved by a linear gradient of 0–100% eluant B mixed with eluant A in 50 min at 1.0 mL/min. The column effluent was monitored at 280 nm.

Figure S2. Activity of urea-treated PL and EGCG complexes. The concentrations of PL and EGCG were 5 μmol/L and 0.5-2 mmol/L, respectively, in the original solutions. The solutions were incubated in 100 mmol/L PBS (pH7.4) at 37 °C for 45 min. After that, urea solution was added, and the final concentrations of urea, PL, and EGCG were 2 mol/L, 2.5 μmol/L and 0.25-1 mmol/L, respectively. PL activity was measured after dialysis. In the figure, the number 100, 200, 300, and 400 denoted the [EGCG]/[PL] ratio and the capital U meant the mixture was treated by urea solution. The activities of PL alone (PL), PL with urea solution (PL+U), and PL-EGCG mixture without urea (number 100-400) were compared.

Figure S3. Secondary structures of urea-treated PL and EGCG complexes. a, CD spectra of urea-treated PL and EGCG complexes. The concentrations of PL and EGCG were 5 μmol/L and 0.5-2 mmol/L, respectively, in the original solutions. The solutions were incubated in 100 mmol/L PBS (pH7.4) at 37 °C for 45 min. After that, urea solution was added, and the final concentrations of urea, PL, and EGCG were 2 mol/L, 2.5 μmol/L and 0.25-1 mmol/L, respectively. Before measurement, solutions were diluted 2.5-folds to meet the limit of detection. b, the contents of secondary structure of urea-treated PL and EGCG complexes.

Figure S4. SDS-PAGE of PL-EGCG mixture. The concentrations of PL and EGCG were 2.5 μmol/L and 0.25-1 mmol/L, respectively. The solutions were incubated in 100 mmol/L PBS (pH7.4) at 37 °C for 45 min before measurement. The [EGCG]/[PL] ratios from panel 1 to 4 were 0, 100, 200, and 400, respectively.

Table S1. Absorbance of PL-EGCG mixture at 500 nm (A500). The concentrations of PL and EGCG were 2.5 μmol/L and 0.25-1 mmol/L, respectively. Experiments were done in 100 mmol/L PBS (pH7.4) at 37 °C. The A500 of EGCG alone (1 mmol/L) and PL alone were compared.

**Figure S1**

**
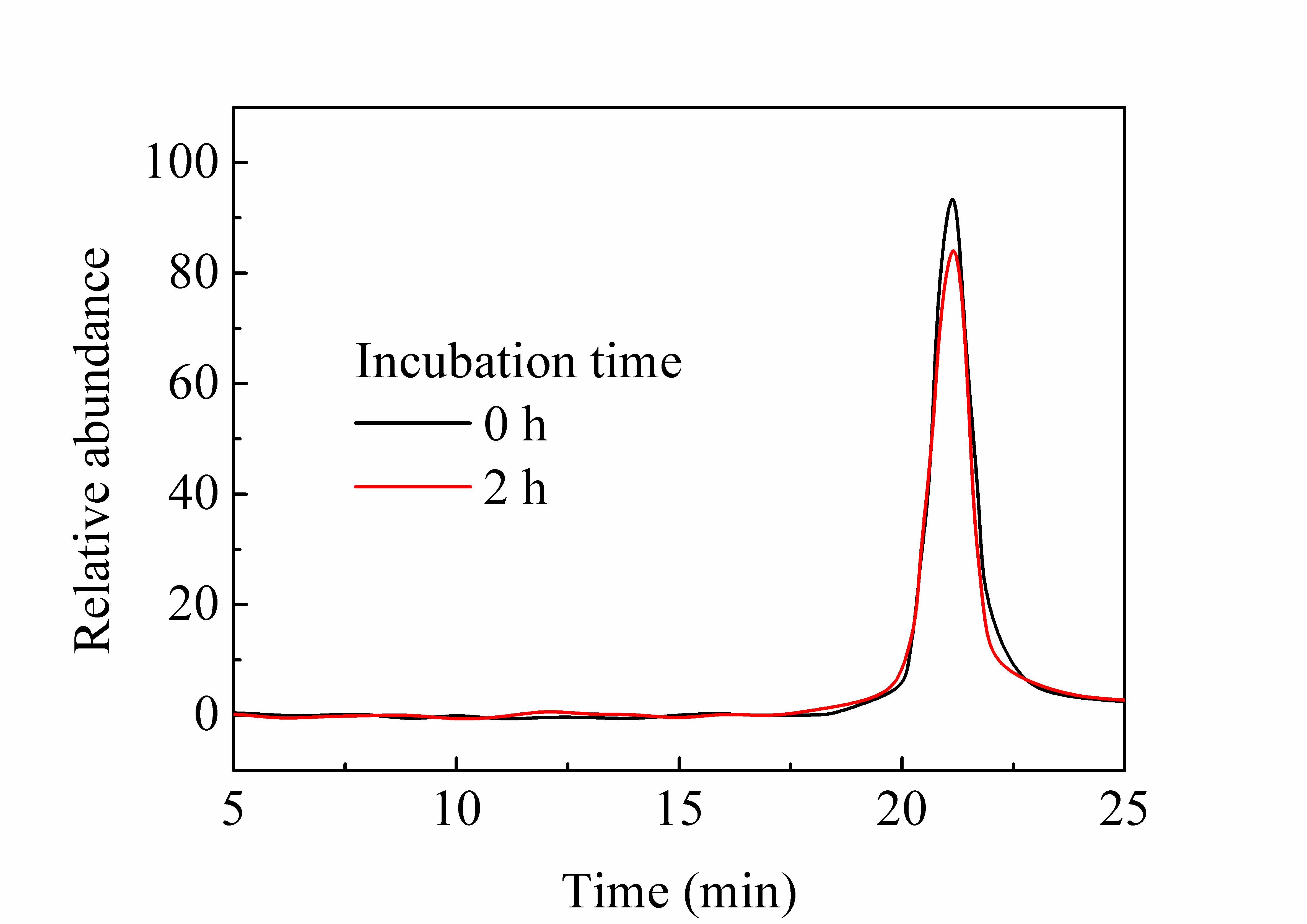
**

**Figure S2**

**
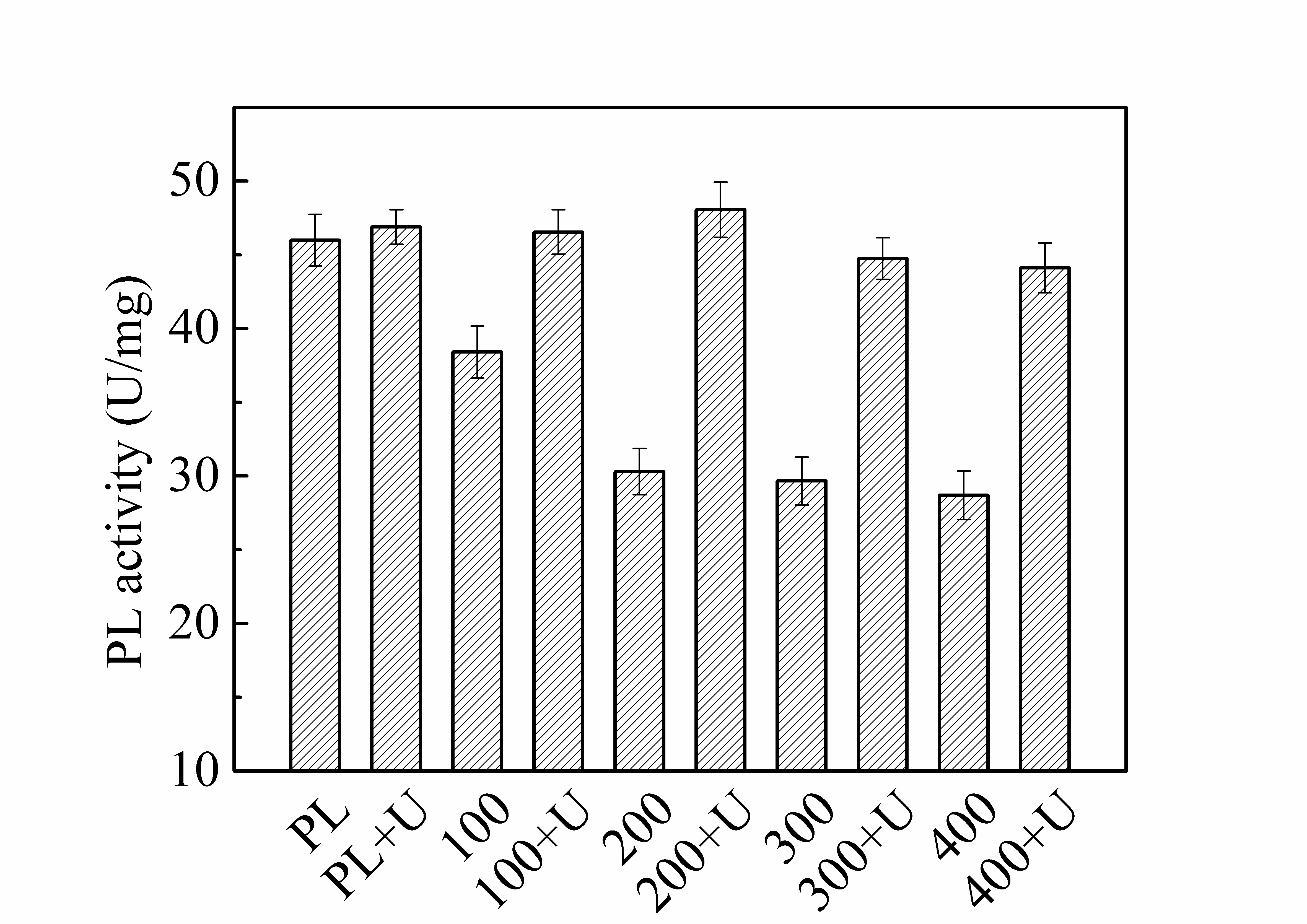
**

**Figure S3**

**a**


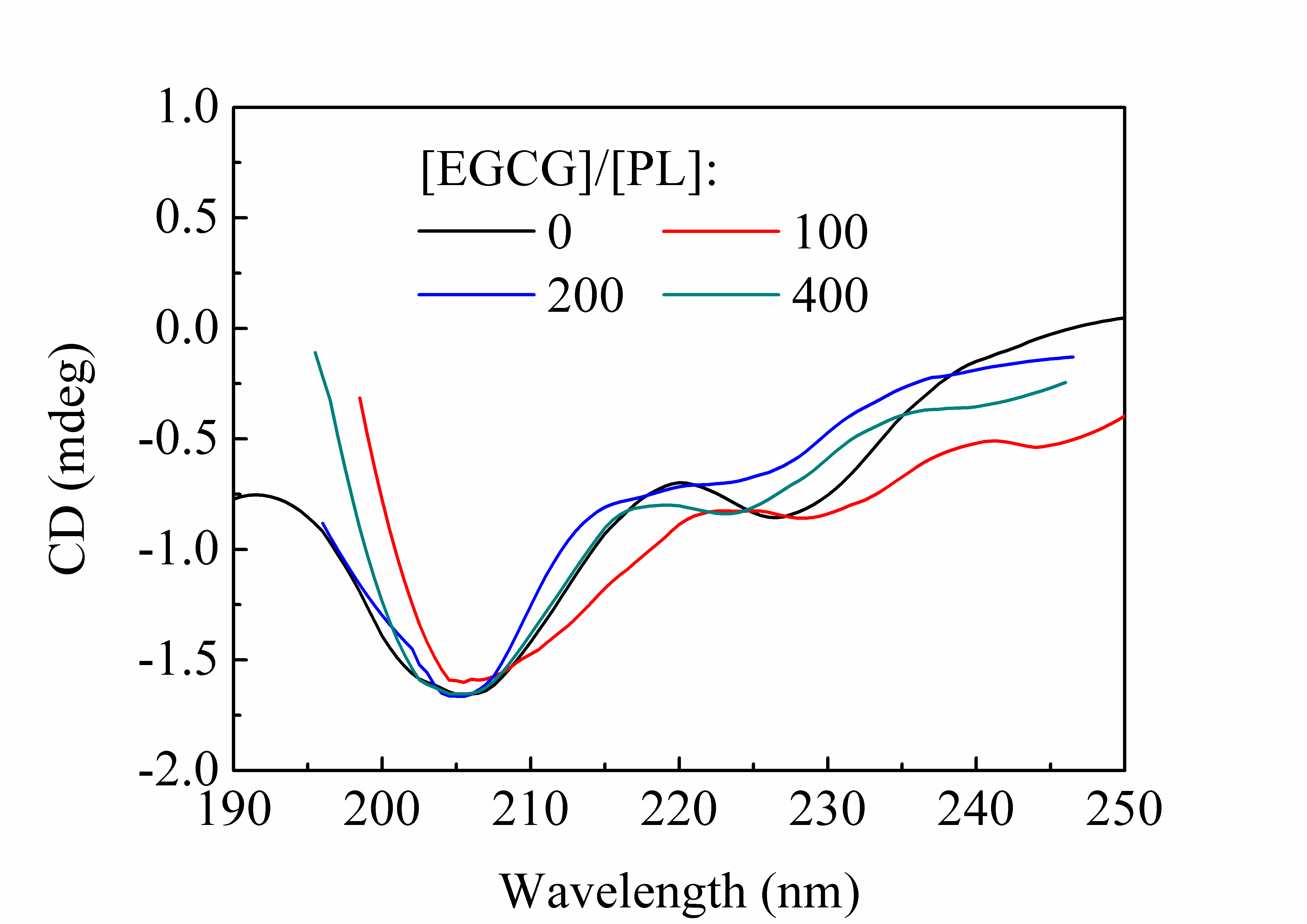


**b**

| *r*=[EGCG]/[PL] | Helix (%) | Sheet (%) | Turn (%) | Unordered (%) |
| --- | --- | --- | --- | --- |
| 0 | 13.7 | 32.5 | 20.5 | 33.3 |
| 100 | 14.8 | 33.2 | 19.4 | 32.6 |
| 200 | 12.1 | 33.7 | 22.5 | 31.7 |
| 400 | 11.3 | 34.5 | 22.9 | 31.3 |

**Figure S4**


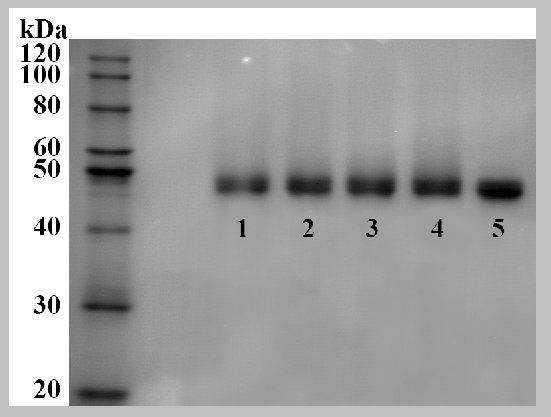


Table S1

| Incubation time (min)  *r*=[EGCG]/[PL] | 0 | 15 | 30 | 45 | 60 |
| --- | --- | --- | --- | --- | --- |
| EGCG alone | 0.0014 | 0.0015 | 0.0017 | 0.0015 | 0.0018 |
| 0 (PL alone) | 0.0010 | 0.0003 | 0.0013 | 0.0007 | 0.0010 |
| 100 | 0.0040 | 0.0040 | 0.0039 | 0.0042 | 0.0045 |
| 200 | 0.0080 | 0.0120 | 0.0129 | 0.0144 | 0.0151 |
| 300 | 0.0183 | 0.0281 | 0.0306 | 0.0337 | 0.0368 |
| 400 | 0.0307 | 0.0307 | 0.0545 | 0.0614 | 0.0660 |
